# Smart Hesperidin/Chitosan Nanogel Mitigates Apoptosis and Endoplasmic Reticulum Stress in Fluoride and Aluminum-Induced Testicular Injury

**DOI:** 10.1007/s12011-023-03991-8

**Published:** 2023-12-13

**Authors:** Nora S. Deiab, Ahmad S. Kodous, Mohamed K. Mahfouz, Alshaimaa M. Said, Mohamed Mohamady Ghobashy, Omayma A. R. Abozaid

**Affiliations:** 1 Biochemistry and Molecular Biology Department, Faculty of Veterinary Medicine, Benha University, Benha, Al Qalyubiyah Egypt; 2 Radiation Biology Department, National Center for Radiation Research and Technology (NCRRT), Egyptian Atomic Energy Authority, P.O. Box 13759, Cairo, Egypt; 3 Department of Molecular Oncology, Cancer Institute (WIA), P.O. Box 600036, 38, Sardar Patel Road, Chennai, Tamilnadu, India; 4 Radiation Research of Polymer Chemistry Department, National Center for Radiation Research and Technology (NCRRT), Egyptian Atomic Energy Authority (EAEA), Cairo, Egypt

**Keywords:** Hesperidin, Nanogel, Aluminum, Fluoride, Testicular damage

## Abstract

Fluoride and aluminum are ubiquitous toxic metals with adverse reproductive effects. The citrus flavonoid hesperidin has protective activities but poor solubility and bioavailability. Nanoparticulate delivery systems can improve flavonoid effectiveness. We conducted this study to prepare a pH-responsive chitosan-based nanogel for hesperidin delivery and evaluate its effectiveness against sodium fluoride (NaF) and aluminum chloride (AlCl_3_) induced testicular toxicity in mice. The nanogel was synthesized using 2 kGy gamma irradiation, enabling a size under 200 nm and enhanced hesperidin release at pH 6 matching testicular acidity. Male mice received 200 mg/kg AlCl_3_ and 10 mg/kg NaF daily for 30 days. Hesperidin nanogel at 20 mg/kg was administered orally either prophylactically (pretreatment) or after intoxication (posttreatment). The results showed that AlCl_3_ + NaF induced severe oxidative stress, hormonal disturbance, apoptosis, and endoplasmic reticulum stress, evidenced by significant changes in the studied parameters and testicular histological damage. Hesperidin nanogel administration significantly inhibited oxidative stress markers, restored luteinizing hormone (LH), follicle-stimulating hormone (FSH), and testosterone levels, and alleviated tissue damage compared to the intoxicated group. It also downregulated the expression level of pro-apoptotic genes Bax, caspase-3, caspase-9, and P38MAPK, while upregulating the expression level of the anti-apoptotic BCL2 gene. Endoplasmic reticulum stress sensors PERK, ATF6, and IRE-α were also downregulated by the nanogel. The chitosan-based nanogel enhanced the delivery and efficacy of poorly bioavailable hesperidin, exhibiting remarkable protective effects against AlCl_3_ and NaF reproductive toxicity. This innovative nanosystem represents a promising approach to harnessing bioactive phytochemicals with delivery challenges, enabling protective effects against chemical-induced testicular damage.

**Graphical Abstract:**

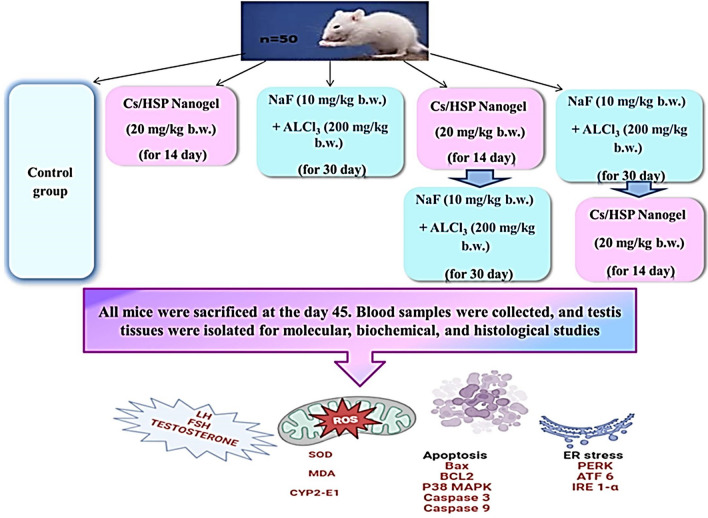

## Introduction

Aluminum (Al) and fluoride (F) are among the most widely distributed toxic metals in the environment. F is the second-largest contaminant in drinking water and one of the most crucial air pollutants [1, 2]. Fluorosis is an endemic disease at least in 25 countries and now is known to be a global challenge. Fluorosis belts are worldwide in distribution; known fluorosis belts include one that stretches from Syria through Jordan, Egypt, Libya, Algeria, Sudan, and Kenya [3]. Recent studies on environmental poisoning have revealed that Al and F may be fundamental risks to humans, plants, and animals [4, 5]. Human exposure to both elements is inestimable, principally through food, breathing contaminated air, medicine-containing Al and F compounds, beverage packaging, toothpaste, and drinking water [6, 7]. In the stomach, F and Al would form fluoro-aluminum complexes (AlF_3_) that exhibit increased transport into the bloodstream [8].

F and Al toxicity is closely linked with a wide array of toxic effects on cellular metabolism, such as alterations in gene expression, suppression of protein synthesis, and DNA damage [9–11]. In synergy with Al, F acts as a false signal in the G protein cascades and affects the regulation of many fundamental processes such as cell metabolism, cytoskeleton protein assembly, energy transduction, cell differentiation, aging, and apoptosis [5]. Aluminu-fluoride complex (AlF_4_^−^) acts as a phosphate analog and stimulates the cellular heteromeric G-proteins; because of its structural similarity with the phosphate group, AlF_4_^−^ is tetrahedral and the Al-F bond length is very similar to the P-O bond length [12].

The endoplasmic reticulum (ER) is a vital organelle that plays a key role in the synthesis of membrane and secreted proteins. A variety of physiological statuses or environmental stimuli can disturb the ER homeostasis, and lead to the accumulation of misfolded and unfolded protein in the ER lumen, a condition referred to as ER stress. To cope with the ER stress’s deleterious effects, cells have evolved the unfolded protein response. However, prolonged ER stress can lead to cell death and is implicated in several diseases [13]. Moreover, recent available studies have demonstrated that redox imbalance can induce ER stress [14, 15]. Previous studies have reported a link between testicular toxicity and endoplasmic reticulum stress [16, 17].

Several studies have shown that hesperidin (hesperetin-7-O-rutinoside), a bioactive citrus flavonoid, can possess antioxidant, anti-inflammatory, hypolipidemic, neuroprotective, radioprotective, anti-epileptic, anti-depressant, anti-carcinogenic, anxiolytic, and hypoglycemic properties [18–20]. Moreover, hesperidin has been reported to alleviate metal-induced toxicity [21, 22], and the testicular protective effect of hesperidin has been established in different studies [23–26]. Recent studies showed that hesperidin can protect against fluoride-induced hepatorenal toxicity [27], as well as cardiotoxicity [28], neurotoxicity [29], and testicular toxicity [30].

Unfortunately, despite its valuable pharmacological and biological activities, hesperidin’s clinical use is extremely restricted; which is attributed to its poor water solubility and poor oral bioavailability. Its bioavailability is estimated to be about 20% [31]. Several approaches have been developed to overcome this obstacle. One approach is the use of nanoparticulate delivery systems which are highly chosen to increase the aqueous solubility of hydrophobic drugs such as hesperitin [32]. Recently, natural polymers for drug delivery have gained great attention in this regard. Chitosan (Cs), the second most abundant naturally occurring polysaccharide, has been studied to find out how well its biodegradability, biocompatibility, safety, muco-adhesive properties, and specificity of targeting [33, 34]. Unlike other natural polymers, the cationic charge possessed by chitosan, besides a large number of active groups (hydroxyl and/or amine groups), is accountable for imparting interesting physical and chemical properties. Considering these unique characteristics, chitosan has been studied as an ideal polymer for hydrogel-based biomedical applications [35], which holds both interesting properties of chitosan and the advantages of hydrogels.

Consequently, this study aimed to fabricate a pH-sensitive nanogel based on poly (acrylamide/acrylic acid) and chitosan by γ radiation to overcome limitations of hesperidin, and then we evaluated the possible effect of this smart nanogel against Al and F-induced testicular toxicity.

## Materials and Methods

### Chemicals

Aluminum chloride (AlCl_3_) was purchased from Al-gomhoria Co. (Cairo). All reagents, monomers of acrylamide (AAm) and acrylic acid (AAc), dimethyl sulfoxide (DMSO), and sodium fluoride were purchased from Sigma-Aldrich Co.. Hesperidin (HSP) (95%) was purchased from Al-dawlya Co. and chitosan (Cs) was obtained from Techno-Gen, Gisa, Egypt.

### Irradiation Process

Gamma irradiation of polymer/monomer in aqueous solution was carried out in a 60 Co Gamma (γ) cell instruction at the National Center for Radiation Research and Technology (NCRRT), Egyptian Atomic Energy Authority. The irradiation dose was carried out at a dose of 0.732 kGy/h.

### Radiation Synthesis of pH-Responsive Chitosan-Based Poly (Acrylamide/Acrylic Acid) Hesperidin (Cs/P(AAc/AAm)/HSP) Nanogel

The solution of Cs / HSP was prepared by dissolving 300 μg of HSP and 100 mg of Cs in 5 ml of DMSO/0.1 M acetic acid solvent. Stir the mixture vigorously using a high-speed homogenizer at 1500 rpm for 30 min (label this solution as “A”). Second, the comonomers solution of AAm and AAc was prepared by dissolving 0.05 gm of AAm and 0.5 ml of AAc in 10 ml of 0.1 M HCl solution. The pH of the homogenized solution of (AAm/AAc) monomers mixture was obtained at 1.2. The comonomers of (AAc/Am) were subjected to γ irradiation at a dose of 2 kGy to start the polymerization reaction. The obtained nanogel of p (AAc/AAm) was labeled as “B.” The pH value of this dispersion solution is ranged from 1 to 2. After that, a drop of NaOH (0.01 M) was added to dispersion solution (B) until pH reached 8 at this pH the nanogel complex (AAc/AAm) opened. Then, solution (A) was added dropwise to a solution (B) and kept pH ranging from 5 to 6. Finally, acetic acid (0. 1 M) was added to the mixture solution (A + B) to adjust the pH to 4. This step ensures that the final suspension solution of chitosan-based p(AAc/AAm)/hesperidin (Cs/p(AAc/AAm)/HSP) is at the desired pH. Preserve the obtained suspension solution at a temperature of 4 °C.

### Characterization of Cs/P(AAc/AAm)/HSP Nanogel

The particle size and zeta potential of Cs/P(AAc/AAm)/HSP nanogel were determined by dynamic light scattering (DLS) instrument using a (Horiba Scientific SZ-100, Japan) operating with at 90 light scattering angle and temperature of 25 °C. HSP release at different pH was investigated by ultraviolet–visible (UV/Vis) spectroscopy (Thermo Fisher Scientific Inc., USA) at 200 to 700 nm. The nanogel size and morphology were investigated by a transmission electron microscope (TEM) using Talos Arctica Cryo-transmission electron microscope at 200 keV.

### Determination of Acute Toxicity (LD50) of Cs/P(AAc/AAm)/HSP Nanogel in Rats

The LD50 is often an early step in determining and estimating chemical hazardous. First, we gave different doses of the nanogel ranging from 12.5 to 100 mg/kg orally to groups of rats each day. Then we observed the mice for any signs of toxicity or mortality. Based on the doses given and the number of rats that died at each dose, the LD50 was calculated [36, 37], this is the dose at which 50% of mice are expected to die. We used a standard formula that considers the lowest dose that killed mice (Dm), the difference between doses (a), the average mortality at each dose (b), the number of rats per group (N), and the sum of (a × b) is ∑.

$$\mathrm{LD}50=\mathrm{Dm}-[\sum (\mathrm{a}\times \mathrm{b})/\mathrm{N}]$$

From this analysis, a dose of 20 mg/kg was safe and did not cause toxicity or mortality in the rats. So 20 mg/kg was selected as the nanogel dose to give to the mice in the subsequent experiments since it was well below the estimated LD50.

### Experimental Design

The care and use of laboratory animals were carried out according to the protocols approved by the Institutional Animal Ethical Committee following the international guidelines for animal experimentation. Additionally, the present study was approved by the Institutional Animal Care and Use Committee Research Ethics Board (BUFVTM 12–11-22). Fifty adult male Swiss albino mice at the age of 40–45 days, with an average weight of 25–35 g were purchased from El Nile Pharmaceutical Co. All mice were examined for health status and their room was designed to maintain the temperature at 25 °C, relative humidity at approximately 50%, and 12 h-light/dark photoperiod with free access to standard rodent diet and ad libitum water. The chow diet for the mice was purchased from El-Nasr Co. (Cairo, Egypt) and its composition is shown in Table 1.

**Table 1:** The composition of normal chow diet

Components	Percentage
Protein	23%
Fat	5%
Carbohydrates	54%
Minerals and vitamins	10.5%
Fibers	7.5%

**Fig. 1:**
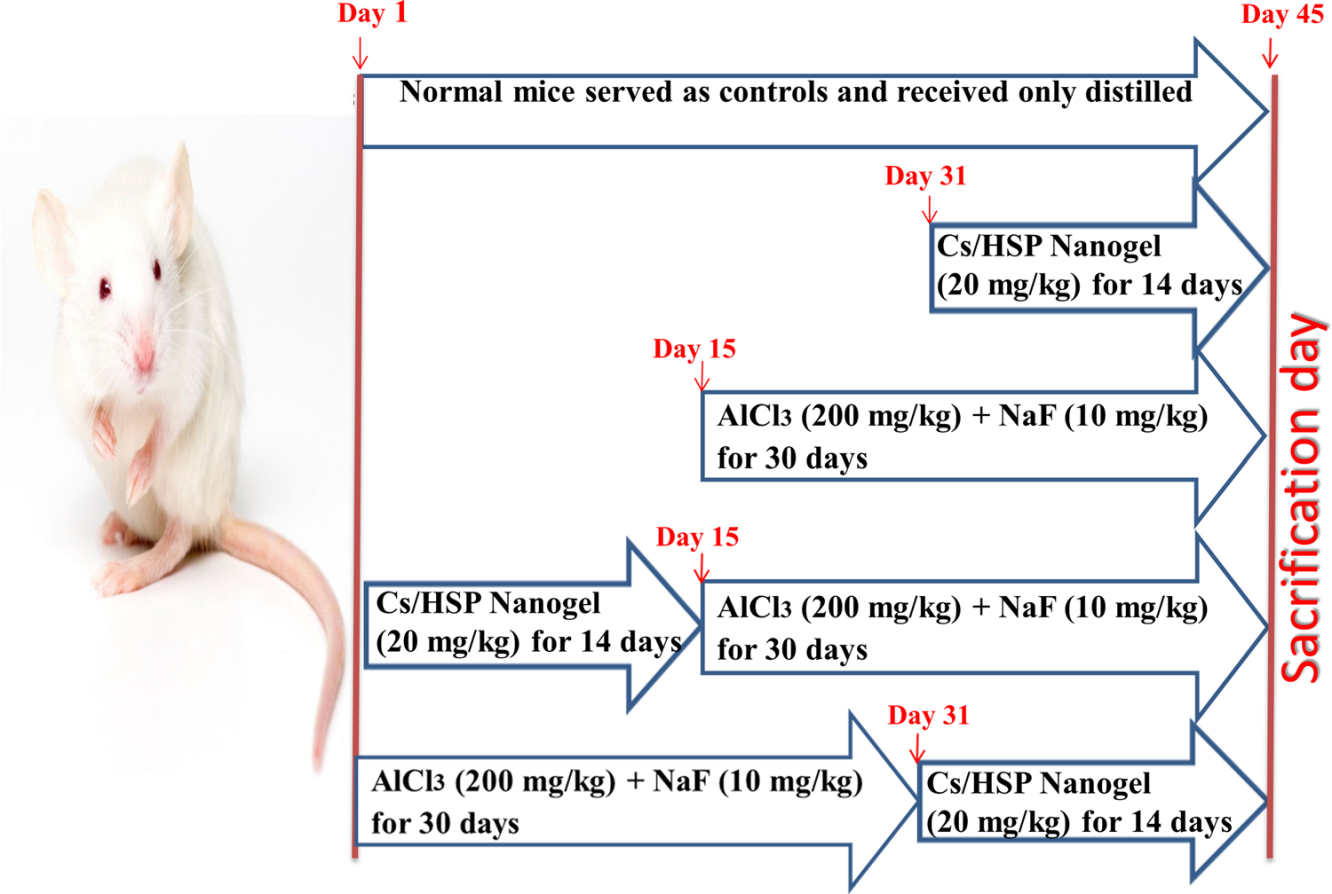
Experimental design

After acclimatization, mice were divided into 5 groups of 10 each as follows:

- **Control group:** Normal mice served as controls and received only distilled water.
- **Cs/P(AAc/AAm)/HSP nanogel group:** mice were orally administered with Cs/P(AAc/AAm)/HSP NANOGEL (20 mg/kg b.w.) daily for 14 successive days.
- **AlCl**_**3**_** + NaF group**: the mice were intoxicated with AlCl_3_ (200 mg/kg b.w.) and NaF (10 mg/kg b.w.), dissolved in distilled water, by oral gavage, daily for 30 days. The doses of NaF and AlCl_3_ were based on the LD 50 of fluoride in male mice (54.4 mg-F/kg b.w.) and of AlCl_3_ (400 mg–Al/kg b.w.) [38].
- **Cs/P(AAc/AAm)/HSP nanogel + (AlCl**_**3**_** + NaF) group “pretreatment”:** mice were pre-treated with Cs/P(AAc/AAm)/HSP Nanogel (20 mg/kg b.w.) for 14 days and then they were intoxicated with AlCl_3_ + NaF for 30 days.
- (**AlCl**_**3**_** + NaF) + Cs/P(AAc/AAm)/HSP nanogel group “posttreatment”:** mice were intoxicated with AlCl_3_ + NaF for 30 days and after that, they were treated with Cs/P(AAc/AAm)/HSP nanogel for 14 days, as shown in following Fig. 1.

After 45 days, the mice were fasted for 12 h, anesthetized by ketamine (24 mg/kg b.w.) intramuscular injection), and sacrificed via decapitation. Blood samples for serum were collected. Testis tissues were excised and divided into two parts: first part was fixed in 10% neutral formalin overnight, embedded in paraffin, and then cut into 4–5-µm sections for histopathological analysis following hematoxylin and eosin (H&E) staining, and the second part used for biochemical and molecular analysis.

### Biochemical Analysis

Superoxide dismutase (SOD) activity and malondialdehyde (MDA) concentration were determined by the colorimetric method as described in the commercial kits (Bio-diagnostic, Egypt). We used Enzyme-Linked Immunosorbent Assay (ELISA) kit obtained from MyBioSource, Inc. San Diego, USA, according to the manufacturer’s instructions, for accurate determination of cytochrome P450 2E1(Cyp2-E1) (Cat. No. MBS165228), luteinizing hormone (LH) (Cat. No. MBS700807), follicle-stimulating hormone (FSH) (Cat. No. MBS2021901), and testosterone (Cat. No. MBS282195). The absorbance at 450.0 nm was determined by using a microplate reader (Bio-Rad model 680, USA).

### Molecular Analysis

Quantitative real-time polymerase chain reaction (qPCR) was used to investigate the changes in mRNA expression for mitogen-activated protein kinase P38 (P38MAPK), BCL2-associated X protein (Bax), B cell lymphoma-2 (BCL2), cysteine-aspartic acid protease-3&-9 (caspase 3&caspase 9), double-stranded RNA-activated kinase (PKR)-like ER kinase (PERK), activation transcription factor 6 (ATF 6), and inositol requiring enzyme-1(IRE 1-α) genes. Pure RNA was extracted from 30 mg of testis tissue using a total RNA Purification Kit following the manufacturer protocol (Thermo Scientific, Fermentas, #K0731). First-strand complementary DNA (cDNA) synthesis was performed using Reverse transcription kits (Thermo Scientific, Fermentas, #EP0451) using template 5 µg RNA. Real-time PCR with SYBR Green was used to measure gene expression. The isolated cDNA was amplified using 2 × Maxima SYBR Green/ROX qPCR Master Mix and gene-specific primers, following the manufacturer protocol (Thermo Scientific, USA, # K0221). The primers used in the amplification are shown in Table 2.The final reaction mixture was placed in a StepOnePlus real-time thermal cycler (Applied Biosystems, Life Technology, USA) included initial step 95 °C for 10 min; 40 cycles at 95˚C for 15 s, 60 °C for 30 s, 72 °C/ 30 s, and finally temperature increased to 95 °C to produce a melt curve. The quantities critical threshold (Ct) of the target gene were normalized with quantities (Ct) of (β-actin), as a housekeeping gene, using the 2^−∆∆^Ct method [39, 40].

**Table 2:** Forward and reverse primers sequence for primers used in qPCR

Gene	Forward primer(/5 ––– /3)	Reverse primer (/5 ––– /3)
Bcl2	ATCGCTCTGTGGATGACTGAGTAC	AGAGACAGCCAGGAGAAATCAAAC
Bax	ACACCTGAGCTGACCTTG	AGCCCATGATGGTTCTGATC
Caspase 3	GGTATTGAGACAGACAGTGG	CATGGGATCTGTTTCTTTGC
Caspase 9	AGCCAGATGCTGTCCCATAC	CAGGAGACAAAACCTGGGAA
P38 MAPK	AGGGCGATGTGACGTTT	CTGGCAGGGTGAAGTTGG
PERK	GAAGTGGCAAGAGGAGATGG	GAGTGGCCAGTCTGTGCTTT
IRF 1-α	TTGACTATGCAGCCTCACTTC	AGTTACCACCAGTCCATCGC
ATF6	GGACCAGGTGGTGTCAGAG	GACAGCTCTGCGCTTTGGG
B actin	AAGTCCCTCACCCTCCCAAAAG	AAGCAATGCTGTCACCTTCCC

### Histopathological Examination

Tissue samples from mice testis were collected and fixed in a 10% neutral buffered formalin solution. The specimens were processed as follows, dehydrated in ascending concentration of ethanol, embedded in paraffin wax, and sectioned at 5-µm thickness. Prepared sections were stained by (H&E). The specimens were evaluated with a light microscope. All histopathological changes were examined under a light microscope [41].

### Statistical Analysis

We used the Statistical Software SPSS (Statistical Program for Social Science) version 20.0 to perform the statistical analysis of data, and then we used one-way ANOVA followed by a post hoc test for multiple comparisons. All data were represented as mean ± standard error (SE) where *P* < 0.05 was used to determine if the SE and difference between means are significant.

## Results

### Characterization of Cs/P(AAc/AAm)/HSP Nanogel

#### Evaluation of the PH Effect on the Size and Zeta Potential of the Obtained Cs/p(AAc/AAm)/HSP Solution

**Fig. 2:**
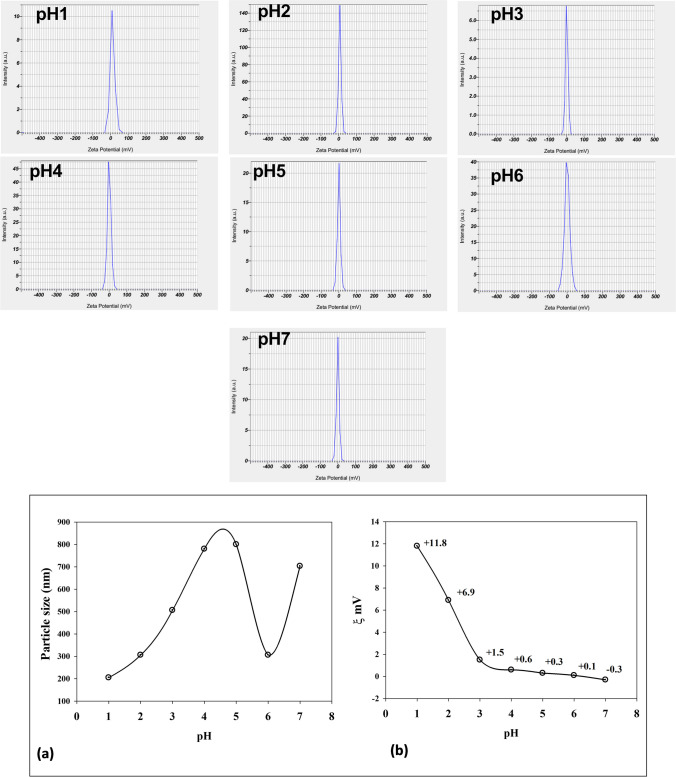
The pH-dependent of (**a**) particle size distribution (nm) and (**b**) zeta potential of Cs/P(AAc/AAm)/HSP samples

Adjustment of the pH of Cs/P(AAc/AAm)/HSP solution from 1 to 7 to select a more stable nanogel/drug complex with a small size that is confirmed by dynamic light scattering (DLS) in Fig. 2. The zeta potential and nanosize properties of Cs/P(AAc/AAm)/HSP were correlated as a function of media pH. Figure 2a illustrates the changes in particle size of the Cs/p(AAc/AAm)/HSP complex across a pH range from 1 to 7. Notably, all data points in the graph confirm that the complex consists of nanoscale particles. At pH 1, the complex exhibits its smallest particle size, measuring approximately 205 nm. As the pH increases, the particle size progressively grows, indicating that the Cs/p(AAc/AAm)/HSP complex is pH-sensitive. Zeta potential reflects the surface charge of nanoparticles and is a critical parameter influencing stability, dispersion, and interactions with biological systems. Figure 2b shows the zeta potentials of the Cs/P(AAc/AAm)/HSP samples were between + 11.8 mV at pH1 and – 0.3 mV at pH 7, indicating a shift towards a negatively charged surface. These changes in surface charge are attributed to the dissociation of acrylic acid (AAc) molecules within the complex, which is pH dependent. In summary, the pH-dependent behavior of the Cs/p(AAc/AAm)/HSP nanogel complex is evident in its particle size and zeta potential variations. The smallest particle size is achieved at pH 1 and 6 offering potential benefits in drug delivery systems. Additionally, the zeta potential changes from positive to near-neutral/negative as pH increases, impacting the complex’s interactions with biological systems.

**Fig. 3:**
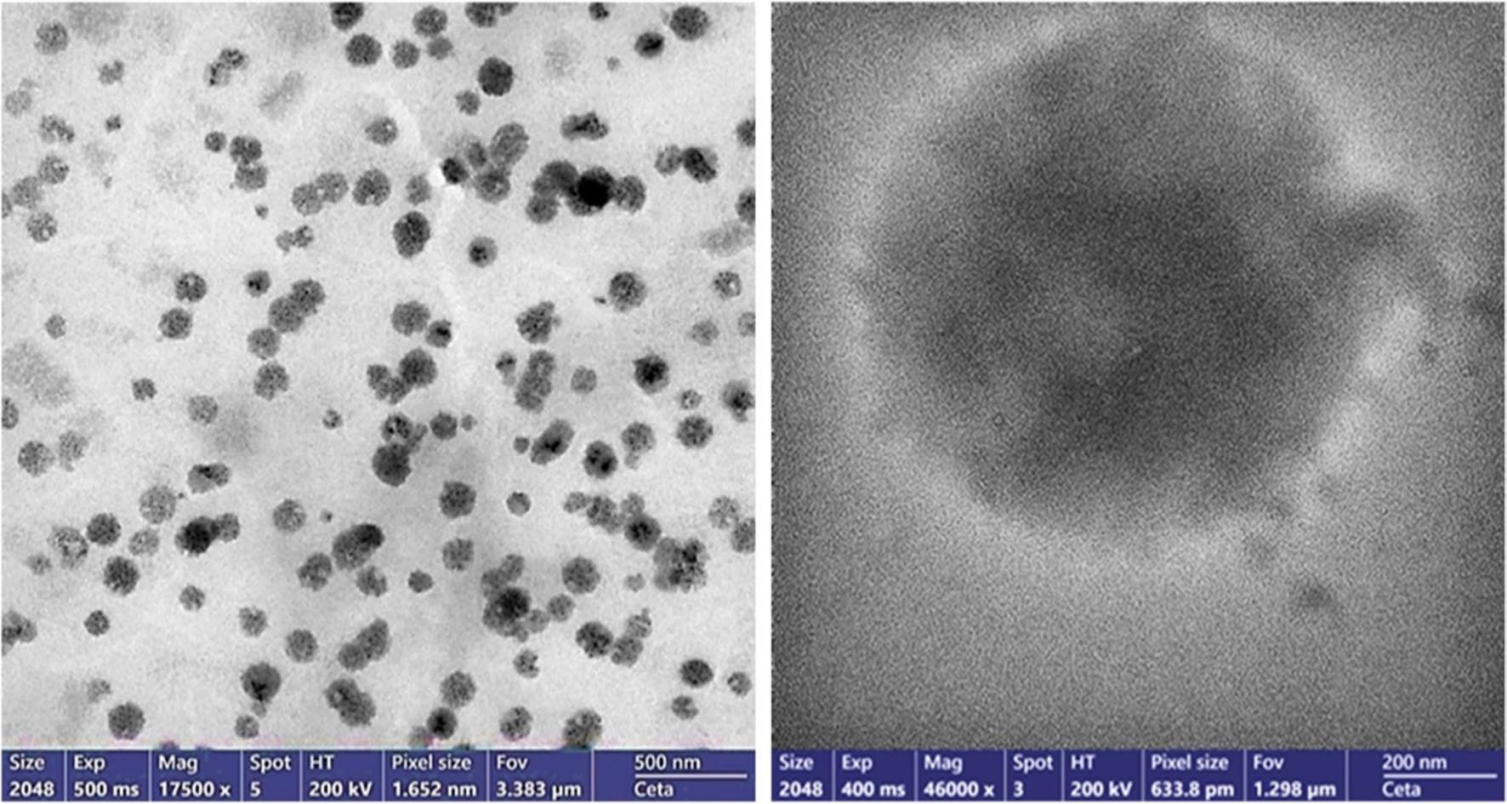
The TEM image of Cs/p(AAc/AAm) nanogel loaded with hesperidin

The transmission electron microscopy (TEM) images, as depicted in Fig. 3 provide critical insights into the morphology and size distribution of the Cs/p(AAc/AAm)/HSP nanogel complex at pH 1. The nanogel complex is visualized as dark dots in the TEM images. Notably, the particle size of the Cs/p(AAc/AAm)/HSP nanogel complex at pH 1 is determined to be less than 200 nm. The distinctive dark dot shape indicates the presence of nanoscale particles within the complex. The TEM images highlight a well-monodispersed distribution of particles, signifying uniformity in size and shape among the nanogel particles within the complex, which makes this complex a promising candidate for applications requiring precise control over size and distribution, such as drug delivery and other nanomedicine applications.

#### Evaluation of the Effect of pH on the Drug Release from the Obtained Cs/P(AAc/AAm)/HSP Nanogel

**Fig. 4:**
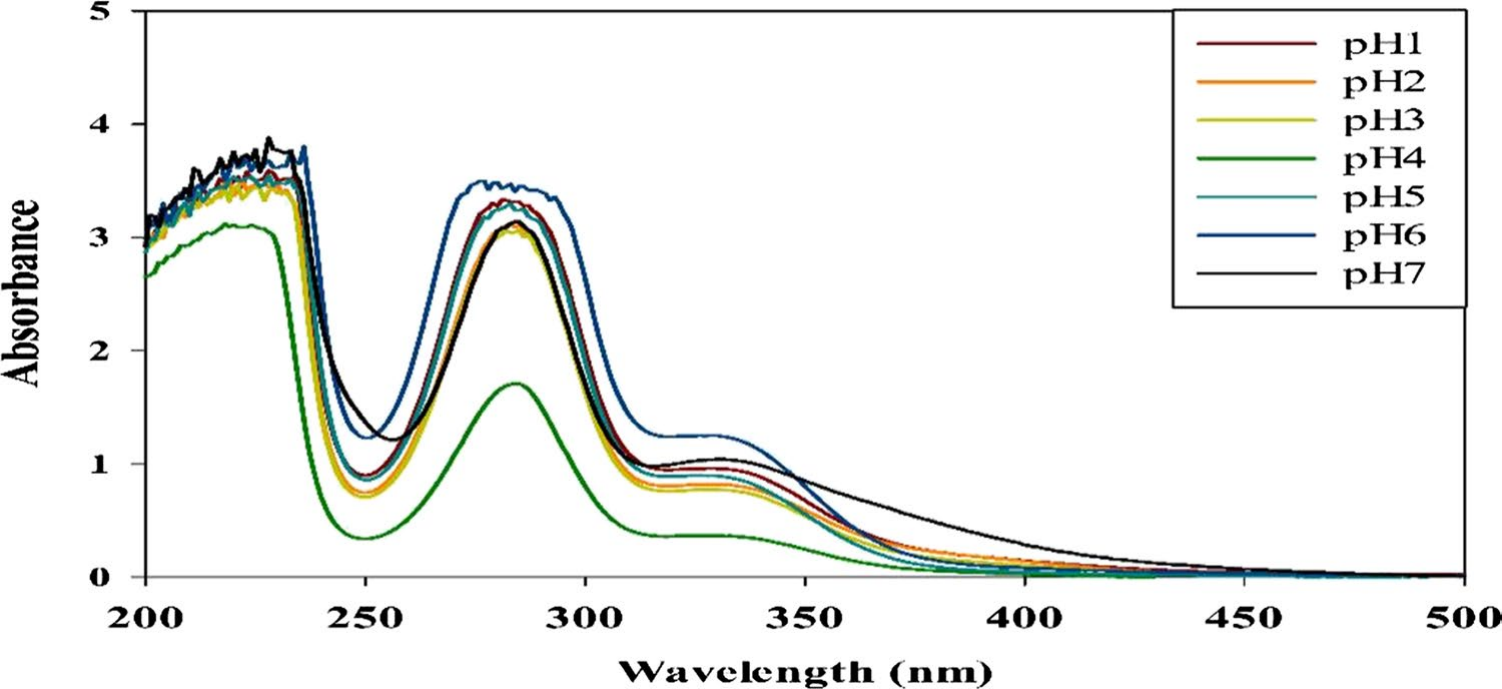
The UV/Vis spectrophotometer of the drug released at different pHs from Cs/P(AAc/AAm)/HSP nanogel

The targeted drug delivery such as pH-responsive controlled release was an additional strategy explored for each pH from 1 to 7. The release system of the nanogel/drug complex solution was investigated by UV/Vis spectrophotometry. As shown in Fig. 4, the data demonstrated relatively higher drug release rates at both pH 1 and pH 6. The small size of Cs/P(AAc/AAm)/HSP at pH1 and pH6 leads to a barrier that alters the retention and release of the drug, suggesting that this pH range might be strategically leveraged for targeted drug delivery applications, particularly those aimed at acidic or slightly alkaline regions within the body. Drug release data in Fig. 4 revealed that the lowest hesperidin release of the obtained Cs/p(AAc/AAm) nanogels was seen at pH 4.

### Histopathological Findings

**Fig. 5:**
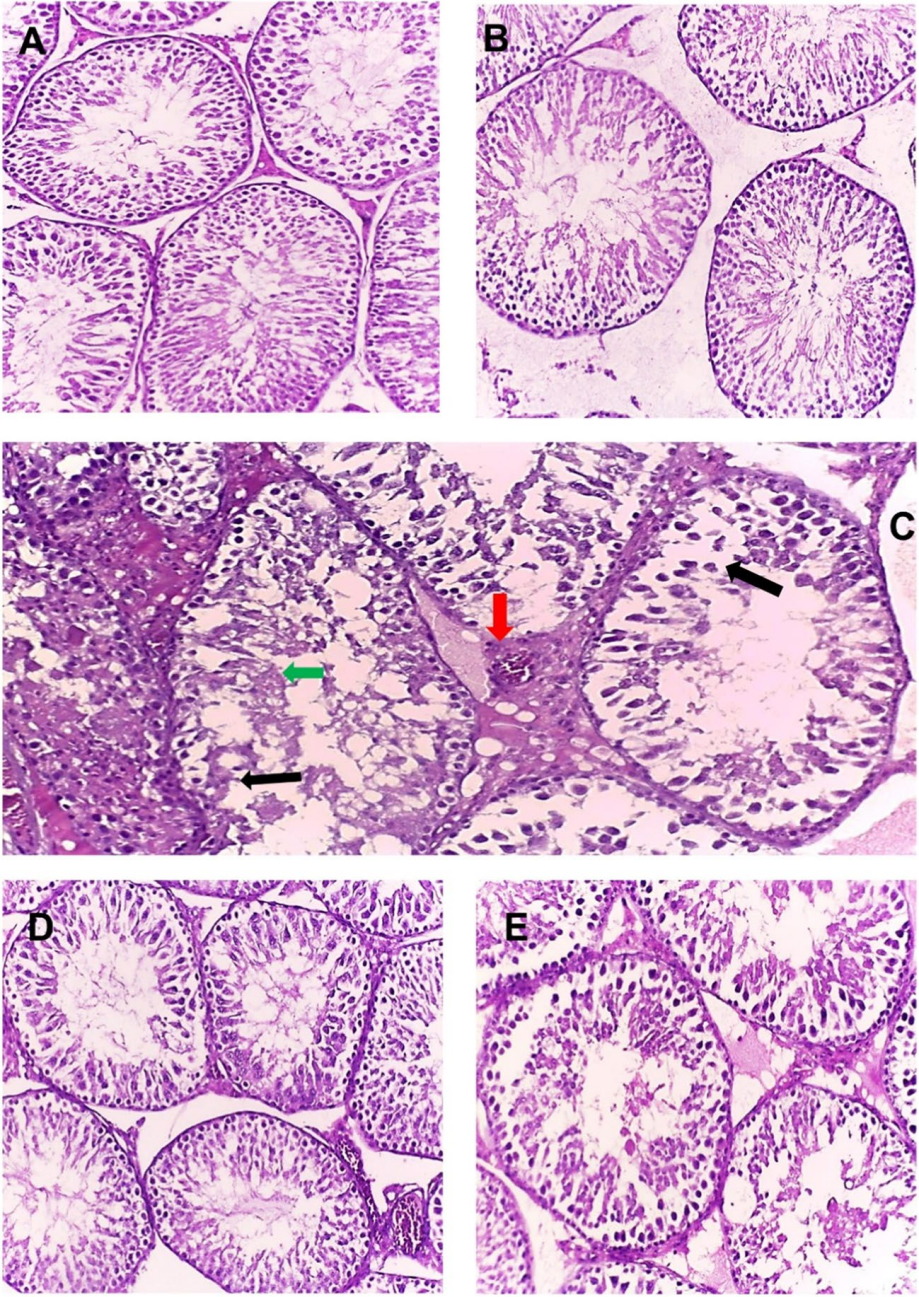
Histopathological photos of testicular tissues (**A**) normal control, **B** Cs/P(AAc/AAm)/HSP nanogel, **C** AlCl_3_ + NaF (intoxicated), **D** pre-treatment, and (**E**) post-treatment (H&E × 200)

Histopathological evaluation of testicular tissues in Fig. 5 revealed distinct differences among the experimental groups. (A) Normal control and (B) Cs/P(AAc/AAm)/HSP groups revealed normal healthy testis architecture with the normal shape of seminiferous tubules, and well-organized Leyding cells, indicative of normal spermatogenesis with minimal cellular damage. The spermatozoa were arranged in groups attached to the inner aspect of the seminiferous tubule lumen. However, in the (C) AlCl_3_ + NaF group, testicular tissues displayed severe pathological changes, including increased apoptosis, obvious disorganized germinal epithelium (black arrow), infiltration in the interstitial area of the seminiferous tubules, congested blood vessels (red arrow), hyperemia, and denudation of spermatogenic cells in the seminiferous tubules lumen (green arrow), with nearly absent sperm bundles, suggesting sever substantial damage to the testis. In the (D) Pre-treatment group, while still partially affected by intoxication, sections restored nearly normal testis histology, with marked signs of recovery. Finally, (E) Post-treatment group, sections showed a partial reduction in apoptosis, mildly disorganized epithelium, mild degeneration, and a decrease in all toxicity findings, indicating a partial protective effect of the Cs/P(AAc/AAm)/HSP nanogel.

#### The Effect of Cs/P(AAc/AAm)/HSP Nanogel on Testis-Oxidative Stress Status

**Fig. 6:**
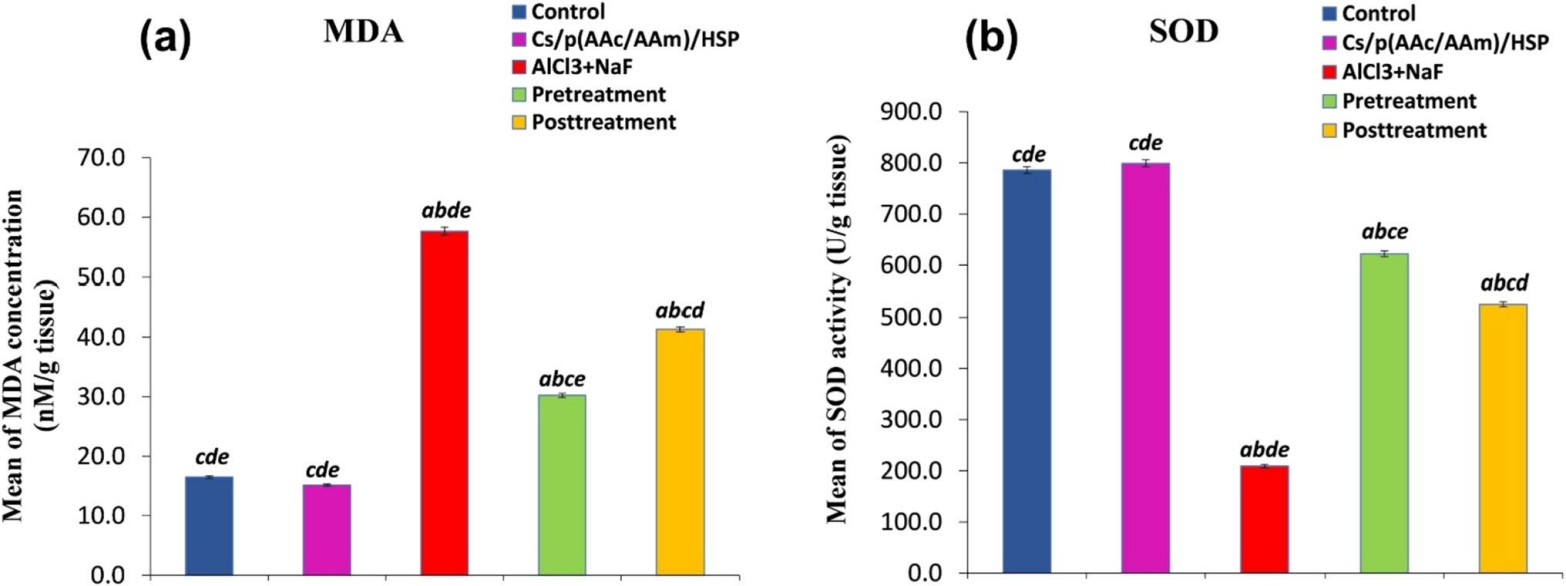
Oxidative stress biomarkers (**a**) MDA concentration and (**b**) SOD activity in all groups. Data were expressed as Mean ± S.E. ^a^*P* < 0.05 versus the control group, ^b^*P* < 0.05 versus the Cs/P(AAc/AAm)/HSP nanogel group, ^C^*P* < 0.05 versus the AlCl_3_ + NaF group, ^d^*P* < 0.05 versus the pre-treatment group, and the ^e^*P* < 0.05 versus the post-treatment group

Figure 6 shows the effects of Cs/P(AAc/AAm)/HSP nanogel on testicular oxidative stress status were profound in the context of AlCl_3_ + NaF-induced toxicity. The intoxicated group exhibited a significant increase (*P* < 0.05) in MDA concentrations, indicating heightened lipid peroxidation, and a significant decrease (*P* < 0.05) in SOD activity, signifying a decrease in antioxidant defense mechanisms, both compared to the control group. However, the oral administration of Cs/P(AAc/AAm)/HSP nanogel, whether as a pre-treatment or post-treatment, effectively normalized these oxidative biomarkers but the pretreatment group showed the best results which signifying the protective antioxidant potential of Cs/P(AAc/AAm)/HSP nanogel. This was evident in the significant (*P* < 0.05) decrease in MDA levels, reflecting a reduction in lipid peroxidation, and the substantial increase in SOD activity, indicative of enhanced antioxidant capacity compared to the intoxicated group. Importantly, the control group and the Cs/P(AAc/AAm)/HSP group did not exhibit significant (*P* < 0.05) differences in the levels of these parameters.

#### The Effect of Cs/P(AAc/AAm)/HSP Nanogel on Testis Endocrinal Markers

**Fig. 7:**
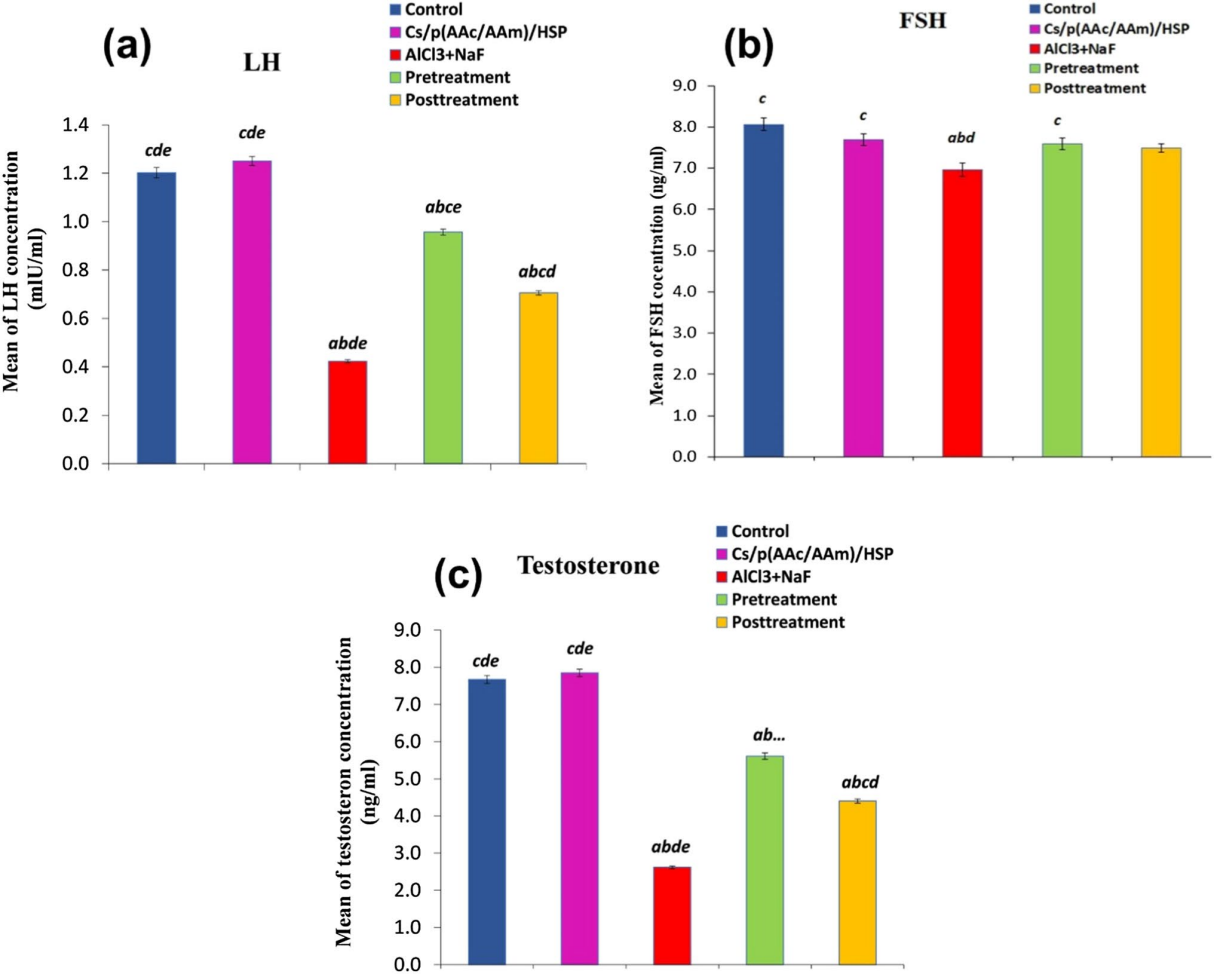
Hormonal assay of endocrinal markers (**a**) LH, **b** FSH, and (**c**) testosterone in all groups. Data were expressed as Mean ± S.E. ^a^*P* < 0.05 versus the control group, ^b^*P* < 0.05 versus the Cs/P(AAc/AAm)/HSP nanogel group, ^C^*P* < 0.05 versus the AlCl_3_ + NaF group, ^d^*P* < 0.05 versus the pre-treatment group, and the ^e^*P* < 0.05 versus the post-treatment group

The impact of Cs/P(AAc/AAm)/HSP nanogel on testicular endocrine markers in the context of AlCl_3_ + NaF-induced toxicity was profound. Exposure to AlCl_3_ + NaF induced a massive decrease in LH, FSH, and testosterone levels (*P* < 0.05), as compared to the control group, reflecting the inhibition of endocrine functions. However, both pre- and post-administration of Cs/P(AAc/AAm)/HSP nanogel effectively mitigated these adverse effects. Notably, this nanogel led to a substantial increase in LH and testosterone levels, with statistical significance (*P* < 0.05) compared to the intoxicated group (Fig. 7a, c respectively). while FSH concentrations decreased significantly in the AlCl_3_ + NaF exposed group compared to the control group, only the Cs/P(AAc/AAm)/HSP nanogel pretreatment group exhibited a significant increase in FSH levels, and there was a slight insignificant (*P* > 0.05) increase in the post-administration group (compared to the intoxicated group) (Fig. 7b). Importantly, there were no statistical differences observed between the control group and the Cs/P(AAc/AAm)/HSP group for any of these endocrine parameters. These results underscore the restorative effect of Cs/P(AAc/AAm)/HSP nanogel on endocrine markers in the testicular tissues, suggesting its potential as a protective and a therapeutic intervention to counteract endocrine disruption induced by AlCl_3_ + NaF.

#### The Effect of Cs/P(AAc/AAm)/HSP Nanogel on CYP2-E1 Levels

**Fig. 8:**
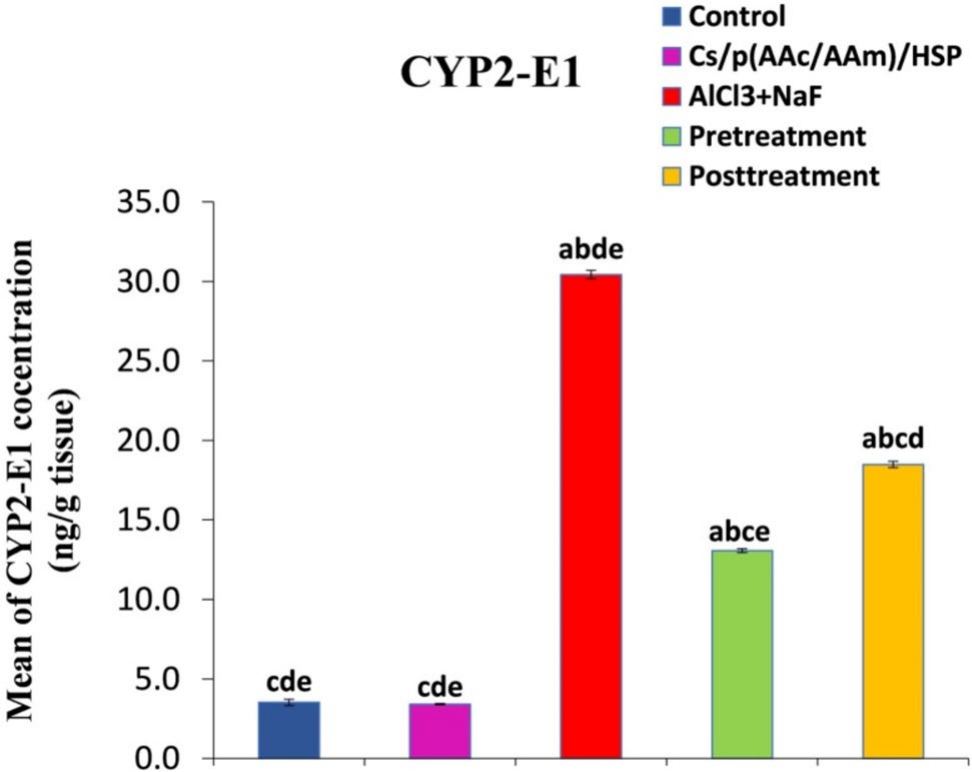
The effect of Cs/P(AAc/AAm)/HSP nanogel on CYP2-E1 levels in all groups. Data were expressed as Mean ± S.E. ^a^*P* < 0.05 versus the control group, ^b^*P* < 0.05 versus the Cs/P(AAc/AAm)/HSP nanogel group, ^C^*P* < 0.05 versus the AlCl3 + NaF group, ^d^*P* < 0.05 versus the pre-treatment group, and the ^e^*P* < 0.05 versus the post-treatment group

Exposure to AlCl_3_ + NaF in normal mice led to a significant increase in CYP2-E1 levels when compared to the control group. However, the administration of Cs/P(AAc/AAm)/HSP nanogel either as a pre- or post-treatment resulted in a significant reduction (*P* < 0.05) in CYP2-E1 levels when compared to the intoxicated group, as demonstrated in Fig. 8. Importantly, there were no significant differences observed between the control group and the Cs/P(AAc/AAm)/HSP group in CYP2-E1 levels. These findings emphasize the efficacy of Cs/P(AAc/AAm)/HSP nanogel in attenuating the elevated CYP2-E1 levels induced by AlCl_3_ + NaF exposure, suggesting its potential in mitigating the toxic effects of CYP2-E1 overexpression in testicular tissues.

#### The Effect of Cs/P(AAc/AAm)/HSP Nanogel on Apoptosis Markers in Testis Tissues

**Fig. 9:**
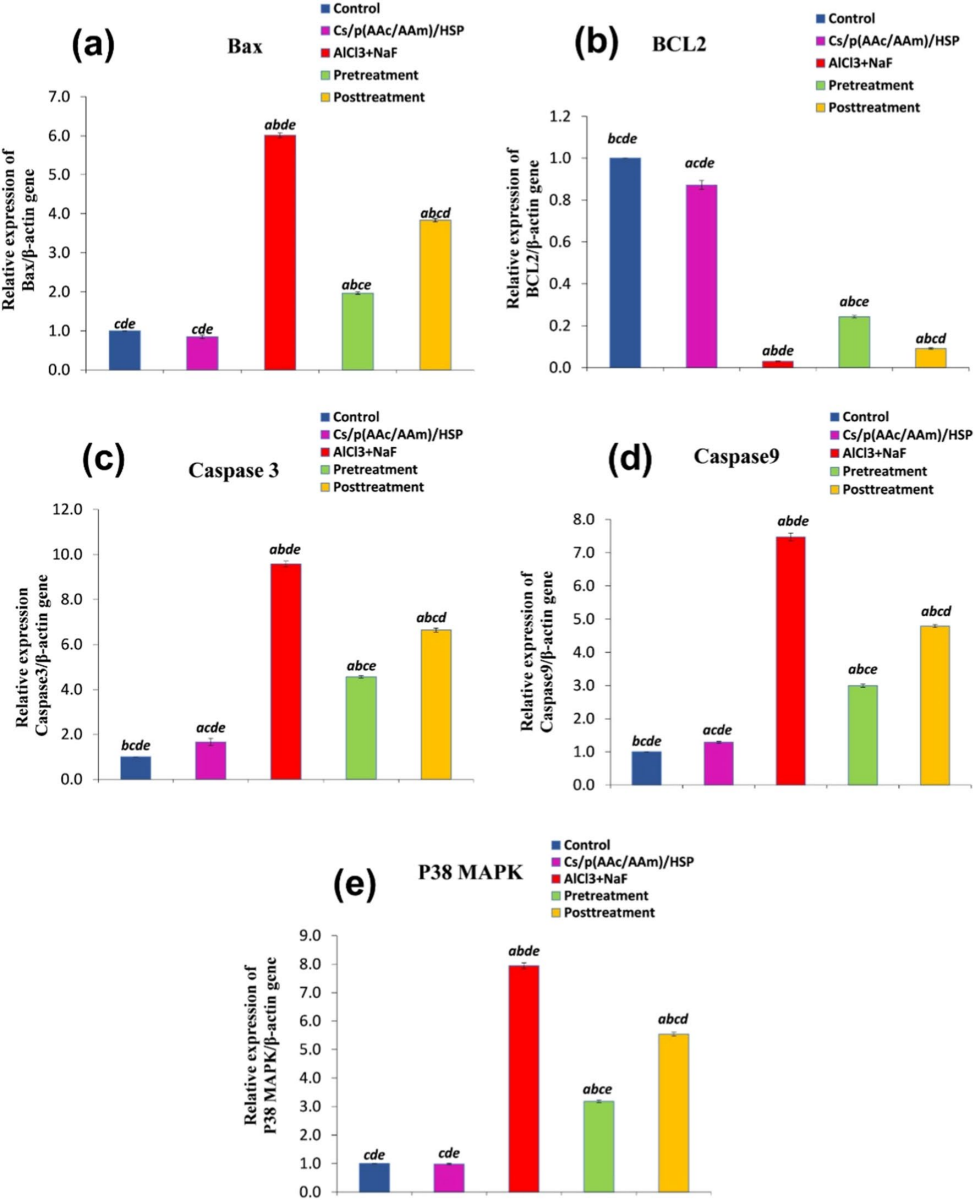
Evaluation of apoptosis markers (**a**) Bax, **b** BCL2, **c** caspase 3, **d** caspase-9, and **e** P38MAPK in all groups. Data were expressed as Mean ± S.E. ^a^*P* < 0.05 versus the control group, ^b^*P* < 0.05 versus the Cs/P(AAc/AAm)/HSP nanogel group, ^C^*P* < 0.05 versus the AlCl_3_ + NaF group, ^d^*P* < 0.05 versus the pre-treatment group, and the ^e^*P* < 0.05 versus the post-treatment group

The oral administration of AlCl_3_ + NaF resulted in a significant (*P* < 0.05) upregulation of pro-apoptotic markers, including Bax, caspase-3, caspase-9, and p38 MAPK, as demonstrated in Fig. 9a, c, d, and e and a marked downregulation of BCL2 relative gene expression (as compared to the control group), as depicted in Fig. 9b. Notably, Cs/P(AAc/AAm)/HSP nanogel administration, whether as a pre-treatment or post-treatment, significantly (*P* < 0.05) inhibited the upregulation of these pro-apoptotic markers (caspase-3, caspase-9, p38 MAPK, and Bax) and elevated the expression of the anti-apoptotic marker BCL2. The pre-treatment group exhibited the most pronounced effect, highlighting the potent anti-apoptotic properties of Cs/P(AAc/AAm)/HSP nanogel “especially as a protective agent” in mitigating apoptosis induced by AlCl_3_ + NaF exposure in testis tissues.

#### The Effect of Cs/P(AAc/AAm)/HSP Nanogel on Testicular Endoplasmic Reticulum Stress Markers

**Fig. 10:**
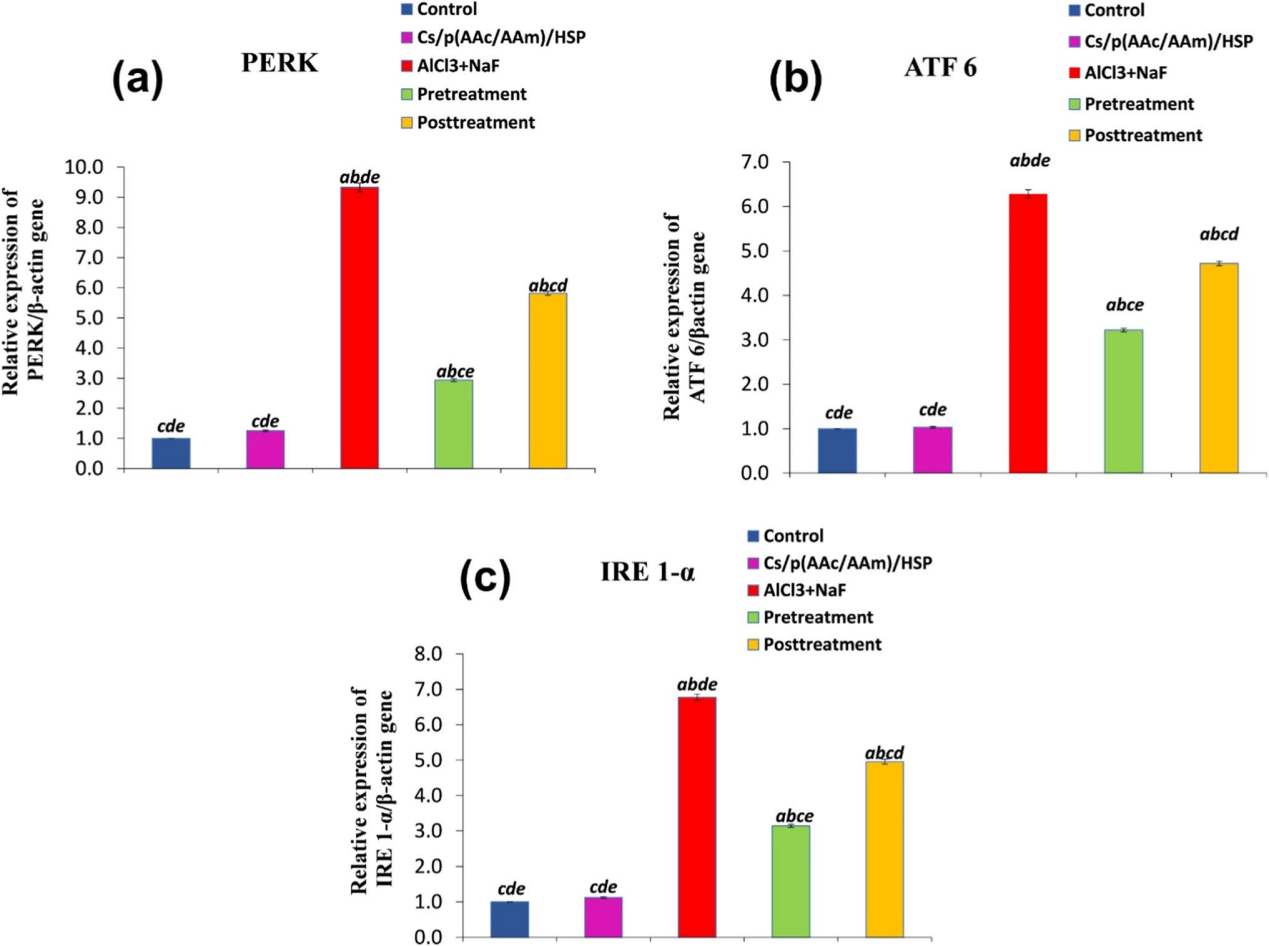
Evaluation of endoplasmic reticulum stress markers (**a**) PERK, **b** ATF 6, and (**c**) IRE 1-α in all groups. Data were expressed as Mean ± S.E. ^a^*P* < 0.05 versus the control group, ^b^*P* < 0.05 versus the Cs/P(AAc/AAm)/HSP nanogel group, ^C^*P* < 0.05 versus the AlCl_3_ + NaF group, ^d^*P* < 0.05 versus the pre-treatment group, and the ^e^*P* < 0.05 versus the post-treatment group

Oral administration of AlCl_3_ + NaF led to a significant (*P* < 0.05) upregulation of ER stress markers, including PERK, ATF 6, and IRE 1-α genes relative expression, as evidenced in Fig. 10a–c, compared to the control group. In contrast, treatment with Cs/P(AAc/AAm)/HSP nanogel resulted in a significant downregulation of the expression of all these ER stress markers relative to the intoxicated group, indicating the potential of the nanogel to mitigate AlCl_3_ + NaF-induced ER stress in testicular tissues. Notably, there were no significant differences in ER stress markers relative expression between the control group and the Cs/P(AAc/AAm)/HSP group.

## Discussion

Our work aimed to assess the impact of AlCl_3_ + NaF exposure on redox status, hormonal markers, specific apoptotic parameters, and the endoplasmic reticulum stress sensors in the testicular tissue of mice. Additionally, we studied the potential benefits of hesperidin. To enhance the solubility and bioavailability of hesperidin, we developed the Cs/P(AAc/AAm)/HSP nanogel. Notably, the pH responsiveness of this nanogel is a significant feature, holding substantial promise for biomedical applications. The pH-dependent behavior of the Cs/P(AAc/AAm) nanogel is characterized by enhanced drug release at pH 6, a crucial feature ensuring efficient drug targeting to the testis, which is known to have a slightly more acidic environment compared to systemic arterial blood [42, 43]. This pH-responsive nanogel can have important implications for drug delivery and treatment strategies in the context of testicular health and beyond. The most intriguing aspect of our study was the discovery that the lowest hesperidin release occurred at pH 4. While the precise mechanisms governing this phenomenon require further investigation, the finding holds significant promise for various applications. In situations where minimal drug release is desired within specific pH environments, such as maintaining drug stability in the gastrointestinal tract, the Cs/p(AAc/AAm)/He nanogel complex at pH 4 could offer exceptional advantages. Further research into the mechanisms responsible for this behavior will undoubtedly shed more light on the full potential of this discovery.

AlCl_3_ + NaF exposure for 30 days caused severe oxidative stress, as evidenced by the decrease in Superoxide Dismutase (SOD) activity, which signifies a compromised defense against harmful reactive oxygen species (ROS). In parallel, there was a notable increase in malondialdehyde (MDA) concentration in testis tissues. MDA is a marker of lipid peroxidation, and its elevated levels further confirm the presence of oxidative damage. The combined effects of lower SOD activity and higher MDA levels underscore the detrimental impact of AlCl_3_ + NaF exposure on the testicular tissues’ redox status. Comparative results reported by Patel and Shahani [44] revealed that exposure to fluoride (F) and aluminum (Al) in mice for longer durations of 45 and 60 days also led to the induction of oxidative stress in the testis and epididymis, manifested by a significant inhibition of catalase (CAT), superoxide dismutase (SOD), and glutathione (GSH). These enzymes play a vital role in the cellular defense against the deleterious effects of ROS. Particularly, SOD serves as the first line of defense by turning the superoxide radicals into less harmful hydrogen peroxide (H_2_O_2_) and molecular oxygen, thus preventing oxidative damage in the cells. ROS induces lipid peroxidation which plays a key role in sperm function deterioration and the pathophysiology of male infertility [45, 46]. Kocak et al. [47] showed that exposure to fluoride (25, 50, and 100 ppm) for 30 days, affects sperm function, triggers testicular oxidative stress, and disrupts the dynamic thiol/disulfide homeostasis, which is an antioxidant defense mechanism that protects cells against oxidative stress.

Interestingly, aluminum (Al), which is often considered a “non-redox active metal,” can indeed exhibit pro-oxidant properties. This occurs through the formation of an aluminum superoxide semi-reduced radical cation, AlO_2_˙^2+^ [48]. Previous studies showed that Al-induced testicular damage is closely related to oxidative stress [49, 50]. In this context, non-redox active metals like aluminum do not readily participate in the electron transfer reactions. However, under certain conditions, aluminum can become involved in redox reactions, generating ROS like superoxide radicals (O_2_^•−^) and hydrogen peroxide (H_2_O_2_). When aluminum reacts in the presence of oxygen, it can form AlO_2_˙^2+^, which is essentially an aluminum superoxide radical. This radical is a type of ROS and can contribute to oxidative damage within biological systems. Understanding the mechanisms by which seemingly non-redox active metals like aluminum can promote oxidative stress is important in assessing their potential impact on health. Additionally, finding ways to mitigate these effects, as our study explores with hesperidin and nanogel administration, is a valuable area of research for improving overall well-being. Furthermore, F affects intracellular redox status, causes intensive nuclear DNA damage, as well as mitochondrial dysfunction, and enhances oxidation of membrane lipids and proteins [51]. Fluoride’s impact on DNA integrity is extensive, leading to both single- and double-stranded DNA damage as a consequence of oxidative stress [52]. This damage to the DNA molecule can manifest as breaks, cross-linking, or alterations in the DNA structure. It is a major cause of the genetic and cellular problems linked to fluoride exposure.

Several studies have reported the efficiency of flavanones against oxidant-induced testicular toxicity. The Cs/P(AAc/AAm)/HSP nanogel pre- and post-treated groups exhibited a significant amelioration of oxidative stress indicators, as evidenced by increased SOD activity, and decreased MDA levels. Worth to mention that, in our study, HSP nanogel showed more effective antioxidant potency when used as a protective than a therapeutic agent.

Our findings are consistent with previous research, affirming the antioxidant potential of hesperidin against fluoride and aluminum-induced testicular toxicity [30, 53]. Oxidative stress can disturb the pivotal male hormone regulators. Noteworthy, normal steroidogenesis produces ROS, mainly by mitochondrial respiration, as well as the steroidogenic cytochrome P450 enzymes catalytic reactions. These ROS, in sequence, have been reported, to inhibit consequent steroid hormone production and damage the spermatozoa mitochondrial membranes [54, 55].

CYP2-E1 is a member of the cytochrome P450 enzymes which are a class of heme-containing enzymes that are implicated in phase I metabolism of different chemicals. CYP2-E1 is not only expressed in liver tissue but also other tissues, including the kidney, lung, pancreas, brain, and testis. It is involved in the metabolic activation of toxins, carcinogens, and several pollutants [56–59]. CYP2-E1 has been implicated in different pathological conditions such as diabetes and cancer, possibly due to its capacity to generate high levels of ROS [60]. To understand CYP2-E1’s precise role in oxidative stress and the reproductive system, it is essential to highlight that some studies have declared the presence of a high-inducible CYP2-E1 isoform in male gonads [58, 61]. In our work, AlCl_3_ + NaF intoxication caused an elevation of CYP2-E1 levels, along with severe oxidative stress, which in tissues like the testis with its high metabolic activity and cell replication may be damaging. In a study by Shayakhmetova et al*.* [62], they demonstrated that the CYP2-E1 elevation in testicular tissues of isoniazid-treated rats resulted in the accumulation of ROS that caused testicular toxicity, spermatogenesis disturbances, and DNA fragmentation. Similar observations had been reported by El-Akabawy and El-Sherif [59], in a study of furan-induced testicular toxicity in rats. Moreover, high levels of CYP2-E1 were recorded in trichloroethylene and acrylamide reproductive toxicity [63–65]. During the last decades, there were efforts to develop drugs that target CYP2-E1 inhibition, and flavonoids, e.g., quercetin, kaempferol, and apigenin, showed promising results [66, 67]. Similarly, in our work, the Cs/P(AAc/AAm)/HSP nanogel pre- or post-treatment attenuated the oxidative stress and reversed the AlCl_3_ + NaF-mediated increase in CYP2-E1 levels. A low hesperidin dose showed an inhibitory effect against CYP2-E1 in fatty liver disease [68] and in thioacetamide-induced hepatotoxicity [69].

In our work, the AlCl_3_ + NaF-treated group showed androgenesis inhibition, marked by a significant decrease in luteinizing hormone (LH), follicle-stimulating hormone (FSH), and testosterone concentration as compared to the control group. This hormonal disturbance is a key indicator of testicular damage and spermatogenesis dysfunction. Comparable results were reported by Das et al*.* [70], who reported that 20 mg NaF/kg/day for 28 days caused marked oxidative stress in rats’ testicular tissue, along with a significant decrease in plasma levels of LH, FSH, and testosterone. Hormonal disturbance was also reported by Shashi and Khan [71], who observed decreased serum testosterone levels, associated with elevated LH and FSH levels in rats that had been exposed to NaF for 20 or 40 days. Zhao et al. [72] suggested that fluoride is an endocrine disrupter that inhibits the testosterone synthesis pathway due to its effect on the activity of the pathway’s key enzymes, e.g., 3β-hydroxysteroid dehydrogenase and 17 β-hydroxysteroid dehydrogenase as well as the levels of the pathway’s main hormones eg., pregnenolone and androstenedione. Our results are in agreement with those of Ige and Akhigbe [73], who reported that 100 mg of AlCl_3_ /kg b.w. decreased LH, FSH, and testosterone levels in male Wister rats. In comparable studies, 300 mg of AlCl_3_ /kg b.w. for 14 days was shown to increase FSH levels and decrease LH and testosterone levels [74]. Sun et al*.* [75] reported that various doses of AlCl_3_ caused a decrease in LH and testosterone without a notable change in FSH levels in male Wister rats.

We hypothesized that the decrease of testosterone in our study may be due to damage to the Leyding cells, which is because of Al and F-induced oxidative stress plus the enhanced ability of Al to cross the blood-testis barrier following oxidative damage and lipid peroxidation that destroys the testis biological membrane and causes spermatogenic cell atrophy. Additionally, former studies reported that several mechanisms are involved in F-reproductive toxicity, including fluoride impact on Sertoli cells autophagy [76], spermatogenesis [77], immune system and apoptosis in testis [78], and the expression profiles of testicular mRNAs and microRNAs [79], that all are synergistically underlying the detrimental effect fluoride on the testis.

Our study revealed that HSP exhibited a substantial impact on hormonal regulation. When administered either as a pre-treatment or post-treatment, HSP effectively elevated LH and testosterone levels (in comparison to the AlCl_3_ + NaF-intoxicated group), signifying its potential role in restoring endocrine functions. Notably, FSH levels were significantly increased only in the pre-treatment group but remained relatively unchanged in the post-treatment group when compared to the intoxicated group. These findings suggest that HSP plays a role in normalizing the levels of key reproductive hormones, with a notable impact on LH, FSH, and testosterone, potentially mitigating the endocrine disruption caused by toxic exposure. These results echo previous research demonstrating HSP’s ability to alleviate oxidative stress and restore LH, FSH, and testosterone hormones to their baseline values in animal models exposed to endocrine disruptors, such as bisphenol [80]. Noshy et al. [81] stated that HSP improves testicular functions via upregulation of the steroidogenesis-related genes. In related studies, HSP showed anti-inflammatory and anti-apoptotic properties, increased levels of LH, testosterone, and FSH, and protected against cyclophosphamide-induced testicular damage by interfering with the hypothalamic-pituitary–gonadal axis [82]. Similar studies reported that hesperidin was effective as a pre-treatment agent against doxorubicin testicular toxicity, with significant improvement in testis lipid peroxidation, and elevation of LH, testosterone, and FSH serum levels [83]. HSP also showed promising results against etoposide [84] and decabromodiphenyl ether [85] induced reproductive toxicity.

Evidence has emerged that oxidative stress-induced apoptosis is a key factor in the toxicity of F and Al [86, 87]. Many studies suggest that F-induced testicular toxicity is attributed to excessive apoptosis [88, 89]. The cysteinyl aspartate specific protease (caspase) family is one of the key proteins in apoptosis regulation. Caspase 9 plays as an initiator caspase which cleaves and activates executioner caspases. After cytochrome c (Cyt C) is released from mitochondria to the cytoplasm, it binds to apoptosis protease activating factor-1(APAF1) and procaspase-9 to constitute an apoptosome, which promotes the activation of caspase-9, which in turn stimulates downstream effector caspases, such as caspase-3 and caspase-7 [90–92]. The involvement of MAP kinases in the F and Al-induced apoptosis and cytotoxicity is proven in many studies [93–97], it is also known to have a pivotal role in regulating spermatogenesis and cell death [98]. In our study, results revealed that rats exposed to AlCl_3_ + NaF showed significant upregulation of pro-apoptotic markers (Bax, caspase-3, caspase-9, and P38MAPK), with subsequently marked downregulation of anti-apoptotic marker (BCL2), suggesting that the intrinsic apoptosis pathway is implicated in testicular apoptosis induced by F and Al. Previous in vivo and in vitro studies showed that F exerts apoptosis through the extrinsic and intrinsic apoptotic pathways, revealing that apoptosis is the core mechanism of fluoride-induced tissue damage [99, 100]. Fluoride exposure triggers apoptosis through the intrinsic apoptotic pathway in fish kidneys [101], rat liver [102], rat kidney [103], oocytes in the female mice ovary [104], female rats thymus [105], NaF-treated renal tubules and MC3T3- E1 osteoblastic cells [106], and pigs hepatocytes [107]. In parallel, previous studies reported that AlCl_3_ administration potentiates cell death in the testicular tissue via upregulation in the levels of apoptotic markers (caspase-3,-9, and Bax), along with downregulation in levels of the anti-apoptotic marker BCL2, which is in agreement with our results [108–110].

In our investigation, the administration of HSP either as a pre-treatment or post-treatment in the context of NaF + AlCl_3_-induced toxicity had a discernible anti-apoptotic effect on testicular tissues. This effect was evidenced by the upregulation of BCL2 gene expression levels, a key anti-apoptotic marker, and the concurrent downregulation of genes associated with apoptosis, including p38MAPK, Bax, and caspase-3&-9, signifying a reduction in apoptotic activity in the testicular tissue. The results underscores the potential of HSP, as a protective or a therapeutic agent, in alleviating apoptosis induced by toxic exposure to fluoride and aluminum, with more pronounced impact in the pre-treatment group. Similar results were reported by Tekin and Çelebi [80], which demonstrated the protective effects of HSP against testicular toxicity induced by bisphenol. In both studies, HSP displayed multifaceted mechanisms of action, including hormonal regulation, attenuation of oxidative stress, and reduction of inflammation. Notably, both studies observed an upregulation of BCL2, a critical anti-apoptotic marker, and a downregulation of Bax, caspase-3, and p38MAPK expression, indicative of HSP’s anti-apoptotic properties. Also, Emre Kızıl et al. [30] demonstrated that HSP helped to reduce NaF-induced testis damage, mitigate oxidative stress, upregulate the anti-apoptotic marker BCL2, and downregulate the proapoptotic markers (caspase-3,-6,-9, and Bax) levels. These consistent results underscore the broad therapeutic potential of HSP in mitigating testicular toxicity and highlight its ability to influence various pathways involved in toxic responses.

Moreover, HSP showed anti-apoptotic activity by regulating the BCL2/Bax ratio against testicular toxicity induced by nickel oxide nanoparticles [81], or cyclophosphamide [82]. Also against neurotoxicity induced by AlCl_3_ [111], and by NaF [29].

The endoplasmic reticulum is a membrane system that is responsible for protein synthesis and processing, and its homeostatic imbalance leads to ER stress. ER stress is triggered by the accumulation of misfolded or unfolded proteins that interfere with normal physiological functions of the cell; hence, the unfolded protein response is activated to recover the ER function and activates the ER stress-transmuting transmembrane protein sensors sequentially, with PERK being the first, followed by ATF6, and IRE1-α being activated last. If the stress is prolonged, or the pro-survival response fails to compensate for the conditions; signaling switches from pro-survival to pro-apoptotic, and apoptotic cell death occurs, which is another endogenous apoptosis pathway besides the mitochondrial pathway [13, 112]. Therefore, many studies have revealed that apoptosis, ER stress, and oxidative stress are interrelated [113, 114]. ER mediates apoptosis via modulation of the downstream pro-apoptotic proteins of the BCL2 family and caspases especially caspase-12 which is found in the ER outer membrane, and stimulates ER-mediated apoptosis via activation of caspase-3&-9 [115], which coincides with our observations. In our study PERK, ATF 6, and IRE 1-α gene expression were significantly upregulated in the AlCl_3_ + NaF intoxicated group, indicative of ER stress. Two different studies on fluoride [116, 117] and aluminum [118] suggested that ER-mediated apoptosis is a critical mechanism for their testicular toxicity. It has been reported that HSP can ameliorate ER stress in fluoride [30], abamectin [119], and paclitaxel [113]-induced testicular toxicity. Our results showed that ER stress was attenuated up on Cs/P(AAc/AAm)/HSP pre- or post-treatment, and these were accompanied by attenuation of oxidative stress, reduction in testicular cell apoptosis, and downregulation of caspase-3&-9 relative expression. Taken together, these results suggest that AlCl_3_ + NaF-induced apoptosis was mediated via ER stress.

While our study provides valuable insights into the effects of Cs/P(AAc/AAm)/HSP nanogel on testicular toxicity in a mouse model, it is important to acknowledge that further in vitro studies are warranted to assess the potential impact of this nanogel on human cells. These in vitro investigations will help validate and expand upon the current findings, allowing for a more comprehensive understanding of the nanogel’s efficacy and safety in human systems. This critical step will contribute to the translational potential of Cs/P(AAc/AAm)/HSP Nanogel for future clinical applications and therapeutic interventions.

## Conclusion

Our study underscores the inevitable exposure to fluoride (F) and aluminum (Al) and the detrimental impact it has on testicular health. The administration of NaF and AlCl_3_ induced severe oxidative stress, mitochondrial and endoplasmic reticulum-mediated apoptosis, perturbed hormonal balance, and extensive degenerative changes in the testicular tissues of male mice. The pre- or post-treatment with Cs/P(AAc/AAm)/HSP Nanogel demonstrated its remarkable potential in ameliorating these adverse effects, with more effective results in the pre-treatment group. This nanogel exhibited strong anti-apoptotic and antioxidant properties, effectively mitigating the damage caused by F and Al exposure. Moreover, it played a regulatory role in restoring normal levels of serum reproductive hormones, making it a promising candidate for protective and therapeutic interventions aimed at improving male fertility and addressing the toxic effects of F and Al in the testis.

## Data Availability

All data obtained from this study are included in the current manuscript.

## Abbreviations

ATF6: Activation transcription factor 6
Bax: BCL2-associated X protein
BCL2: B cell lymphoma-2
Caspase: Cysteine-aspartic acid protease
Cs/P(AAc/AAm): Chitosan-based poly (acrylamide/acrylic acid)
Cyp2-E1: Cytochrome P450 2E1
ER: Endoplasmic reticulum
FSH: Follicle-stimulating Hormone
HSP: Hesperidin
IRE 1-α: Inositol requiring enzyme 1-α
LH: Luteinizing hormone
MDA: Malondialdehyde
P38MAPK: P38 mitogen-activated protein kinase
PERK: Double-stranded RNA-activated kinase (PKR)-like ER kinase
ROS: Reactive oxygen species
SOD: Superoxide dismutase activity
